# Human PAF1 inhibition of HIV-1

**DOI:** 10.1186/1742-4690-10-S1-P39

**Published:** 2013-09-19

**Authors:** Ana Guerrero Alonso, Kelly Marno, William Ogunkolade, Julieta Diaz-Delfin, Eithne O’Sullivan, Áine McKnight

**Affiliations:** 1 Centre for Immunology and Infectious Disease, Blizard Institute, Barts and The London School of Medicine and Dentistry, Queen Mary University of London, London, Greater London, UK

## Background

The Polymerase II-Associated Factor 1 (PAF1) is a cellular protein with essential roles in transcription activation and repression, polyA tail cleavage, regulation of RNA polymerase elongation, regulation of gene expression, cell cycle control and mRNA quality surveillance[1]. It is part of a multicomponent complex together with other cellular proteins including SKI8, LEO1, CDC73, RTF1 and CTR9 [1]. We described that PAF1 has antiviral effect against HIV-1 that manifests early in the replication cycle [2]. Marazzi *et al.* described that PAF1 also inhibits influenza virus infection suggesting that PAF1 could have a broad antiviral activity [3]. Localisation of endogenous PAF1 is mainly in the nucleus but also in the cytoplasm. We propose three hypotheses: that PAF1 acts from the cytoplasm or the nucleus or both.

## Materials and methods

To investigate a link between the antiviral function and cellular localisation of PAF1, human PAF1 mutants were engineered with altered nuclear localization signals. Wild type and mutant PAF1 constructs were characterised by confocal imaging, cellular fractionation and ability to inhibit HIV-1, 2 and SIV. Viral infectivity was measured by quantitative RT-PCR of early and late HIV-1 transcripts as well as focus forming units.

## Results

Results describing the requirements for specific cellular localisations of PAF1 for viral inhibition will be presented.

## Conclusion

Understanding the mechanism by which PAF1 modulates HIV-1 infection could unravel novel drug targets not only against HIV-1 infection but possibly against other viruses.
